# Evaluation of serological cross-reactivity and cross-neutralization between the United States porcine epidemic diarrhea virus prototype and S-INDEL-variant strains

**DOI:** 10.1186/s12917-016-0697-5

**Published:** 2016-04-05

**Authors:** Qi Chen, Joseph T. Thomas, Luis G. Giménez-Lirola, John M. Hardham, Qinshan Gao, Priscilla F. Gerber, Tanja Opriessnig, Ying Zheng, Ganwu Li, Phillip C. Gauger, Darin M. Madson, Drew R. Magstadt, Jianqiang Zhang

**Affiliations:** Department of Veterinary Diagnostic and Production Animal Medicine, College of Veterinary Medicine, Iowa State University, 1800 Christensen Drive, Ames, IA 50011 USA; Zoetis, Kalamazoo, MI USA; The Roslin Institute and The Royal (Dick) School of Veterinary Studies, University of Edinburgh, Roslin, Midlothian, Edinburgh, EH25 9RG UK

**Keywords:** Porcine epidemic diarrhea virus, PEDV, Prototype, S-INDEL, Variant, IFA, Virus neutralization, ELISA

## Abstract

**Background:**

At least two genetically different porcine epidemic diarrhea virus (PEDV) strains have been identified in the United States (U.S. PEDV prototype and S-INDEL-variant strains). The current serological assays offered at veterinary diagnostic laboratories for detection of PEDV-specific antibody are based on the U.S. PEDV prototype strain. The objectives of this study were: 1) isolate the U.S. PEDV S-INDEL-variant strain in cell culture; 2) generate antisera against the U.S. PEDV prototype and S-INDEL-variant strains by experimentally infecting weaned pigs; 3) determine if the various PEDV serological assays could detect antibodies against the U.S. PEDV S-INDEL-variant strain and vice versa.

**Results:**

A U.S. PEDV S-INDEL-variant strain was isolated in cell culture in this study. Three groups of PEDV-negative, 3-week-old pigs (five pigs per group) were inoculated orally with a U.S. PEDV prototype isolate (previously isolated in our lab), an S-INDEL-variant isolate or virus-negative culture medium. Serum samples collected at 0, 7, 14, 21 and 28 days post inoculation were evaluated by the following PEDV serological assays: 1) indirect fluorescent antibody (IFA) assays using the prototype and S-INDEL-variant strains as indicator viruses; 2) virus neutralization (VN) tests against the prototype and S-INDEL-variant viruses; 3) PEDV prototype strain whole virus based ELISA; 4) PEDV prototype strain S1-based ELISA; and 5) PEDV S-INDEL-variant strain S1-based ELISA. The positive antisera against the prototype strain reacted to and neutralized both prototype and S-INDEL-variant viruses, and the positive antisera against the S-INDEL-variant strain also reacted to and neutralized both prototype and S-INDEL-variant viruses, as examined by IFA antibody assays and VN tests. Antibodies against the two PEDV strains could be detected by all three ELISAs although detection rates varied to some degree.

**Conclusions:**

These data indicate that the antibodies against U.S. PEDV prototype and S-INDEL-variant strains cross-reacted and cross-neutralized both strains in vitro. The current serological assays based on U.S. PEDV prototype strain can detect antibodies against both U.S. PEDV strains.

## Background

Porcine epidemic diarrhea (PED), caused by porcine epidemic diarrhea virus (PEDV), was first recorded in England in the early 1970s and has since spread to other European and Asian countries [1]. In North America, PEDV was detected for the first time in the United States (U.S.) in April 2013 [2] and subsequently PEDV was reported in Canada [3] and Mexico [4]. PEDV is an enveloped, single-stranded, positive-sense RNA virus belonging to the order *Nidovirales*, the family *Coronaviridae*, subfamily *Coronavirinae*, genus *Alphacoronavirus* [5]. The PEDV genome is approximately 28 kb in length and includes ORF1a and ORF1b encoding the replicase polyproteins and other opening reading frames (ORFs) encoding four structural proteins [spike (S), envelope (E), membrane (M), and nucleocapsid (N)] and one nonstructural protein NS3B (encoded by ORF3) [1].

In the U.S., a highly virulent PEDV strain (U.S. PEDV prototype strain) was identified during the initial PED outbreaks [2, 6, 7]. Lately, a PEDV variant strain having insertions and deletions (INDEL) in the spike gene compared to the U.S. prototype strain was identified in U.S. swine with mild clinical signs based on field observations [8]. This U.S. PEDV variant strain, also known as S INDEL strain [4], formed a distinct phylogenetic cluster compared to U.S. PEDV prototype strains [4, 8, 9]. One PEDV isolate (PC177) having a 197-aa deletion in the N-terminal S protein was discovered during PEDV isolation in cell culture; however, this PEDV isolate still phylogenetically clustered with the U.S. PEDV prototype strains and was not considered as one of the S-INDEL-variant strains [10]. Marthaler et al [11] reported a ‘third’ strain of PEDV (Minnesota188) in U.S. swine that had 6 nucleotide deletions (2 amino acid deletions) in the spike gene (different from the U.S. S-INDEL-variant strains). However, the PEDV Minnesota188 was genetically very closely related to the U.S. PEDV prototype strains and it is arguable whether it should be called a ‘third’ strain of PEDV in the U.S. The PEDV PC177 and Minnesota188 are probably the mutants of the U.S. PEDV prototype strains. Therefore, there are at least two genetically different PEDV strains currently circulating in U.S. swine: U.S. PEDV prototype strain and S-INDEL-variant strain.

The U.S. PEDV prototype strains have been successfully isolated and propagated in cell culture by several groups [7, 10, 12, 13]. A number of serological assays, including an indirect fluorescent antibody (IFA) assay, a virus neutralization (VN) test, a whole virus-based enzyme-linked immunosorbent assay (ELISA), a recombinant S1 protein-based ELISA, and recombinant nucleocapsid protein-based ELISAs, have been developed for the detection of PEDV-specific antibodies [14–18]. All of these serological assays are based on the U.S. PEDV prototype strains.

In this study, we isolated a U.S. PEDV S-INDEL-variant strain in cell culture. Pigs were experimentally inoculated with a U.S. PEDV prototype strain and the newly isolated U.S. PEDV S-INDEL-variant strain, respectively, to generate strain-specific antisera. Subsequently, the generated swine antisera were subjected to an in vitro evaluation for serological cross-reactivity and cross-neutralization between the two strains. Specifically, 1) PEDV IFA antibody assays (using the prototype and S-INDEL-variant strains as indicator viruses, respectively) and ELISAs (PEDV prototype strain whole virus-based ELISA, PEDV prototype strain S1-based ELISA, and PEDV S-INDEL-variant strain S1-based ELISA) were conducted to evaluate the antibody cross-reactivity of the two U.S. strains; and 2) VN tests using the prototype and S-INDEL-variant strains as indicator viruses were conducted to evaluate the in vitro cross-neutralization of two U.S. strains.

## Methods

### Isolation of U.S. PEDV S-INDEL-variant strain in cell culture

Sixty-eight clinical samples (27 fecal swabs, 24 feces, 13 small intestines and 4 oral fluids), which were tested positive by a PEDV N gene-based real-time RT-PCR [17, 19] at the Iowa State University Veterinary Diagnostic Laboratory (ISU VDL) and confirmed positive for the U.S. PEDV S-INDEL-variant strain but negative for the U.S. prototype strain by a PEDV S gene-based differential real-time RT-PCR (Chen et al., unpublished), were selected to attempt virus isolation in Vero cells (ATCC CCL-81) following previously described procedures [7].

Among the aforementioned 68 clinical samples positive for the U.S. PEDV S-INDEL-variant strain, one small intestine homogenate (with PEDV N gene-based real-time RT-PCR cycle threshold (Ct) value of 16.1) [17, 19] from a pig located in Illinois was inoculated orogastrically into three PEDV-naïve weaned pigs at 3 weeks of age (10 ml per pig). The homogenate used for inoculation was confirmed negative for transmissible gastrointestinal virus (TGEV), porcine rotavirus groups A, B, C and porcine deltacoronavirus (PDCoV) by virus-specific RT-PCRs at the ISU VDL. Rectal swabs and feces were collected from each inoculated pig twice a day and tested by the PEDV real-time RT-PCR on the same day. Once the RT-PCR Ct values of the rectal swabs were <15, the pig was euthanized and necropsied within 24 h. Small intestine tissues and cecum contents were collected for attempting virus isolation in cell culture as previously described [7]. This animal study was performed according to the procedures approved by the Iowa State University Institutional Animal Care and Use Committee (IACUC, approval number 3-14-7766-S).

The whole genome sequence of the U.S. PEDV S-INDEL-variant strain cell culture isolate USA/IL20697/2014 obtained in this study was determined by next-generation sequencing (NGS) technology using an Illumina MiSeq platform as described previously [7]. The PEDV S1 portion sequences of the isolate USA/IL20697/2014 and the clinical sample from which the virus isolate was derived were determined by Sanger sequencing following the previously described procedures [7].

### Generation of antisera against the U.S. prototype and S-INDEL-variant PEDVs

Fifteen 3-week-old pigs, negative for PEDV as confirmed by a real-time RT-PCR on rectal swabs and by IFA antibody assay on sera, were first segregated by weight and then assigned randomly into 3 groups with 5 pigs per group and with similar average weight per group, one group per room. Five pigs within each group were housed together in one room on a solid floor. After acclimation for 3 days, three groups of pigs were orogastrically inoculated with a U.S. PEDV prototype cell culture isolate USA/IN19338/2013 (Pro group) [7], a U.S. PEDV S-INDEL-variant cell culture isolate USA/IL20697/2014 (Var group), and virus-negative culture medium (Neg group), respectively, with virus titers of 10^4^ TCID_50_/ml, 10 ml per pig. Rectal swabs were collected from all pigs daily between 0 and 7 DPI, and then at 10, 14, 21 and 28 DPI, and tested by a PEDV N gene-based quantitative real-time RT-PCR [20] to confirm infection. Serum samples were collected from all pigs at 0, 7, 14, 21 and 28 days post inoculation (DPI) for cross-reactivity and cross-neutralization evaluations. This animal study was performed according to the procedures approved by the Iowa State University IACUC committee (approval number 6-14-7809-S).

Twenty-five serum samples collected at 0, 7, 14, 21, and 28 DPI from the Pro group (Pro antisera), 25 serum samples collected from the Var group (Var antisera), and 25 serum samples collected from the Neg group (Neg antisera), were tested by various serological assays in this study. In addition, one pig antiserum against the European PEDV CV777 strain, one pig antiserum against the TGEV Purdue strain, one pig antiserum against the porcine heamagglutinating encephalomyelitis virus (PHEV), one pig antiserum against the porcine respiratory coronavirus (PRCV), and one pig antiserum against PDCoV were included in this study for evaluations. Antisera against PEDV CV777, TGEV Purdue, and PHEV strains were purchased from National Veterinary Service Laboratory, Ames, IA. Antisera against PRCV and PDCoV were positive control sera obtained from the ISU VDL.

### Indirect fluorescent antibody (IFA) assay

Eighty serum samples were tested by the PEDV prototype strain-based IFA (Pro IFA) and S-INDEL-variant strain-based IFA (Var IFA) following the previously described procedures [20]. The PEDV prototype isolate USA/IN19338/2013 was used as the indicator virus in the Pro IFA assay and the S-INDEL-variant isolate USA/IL20697/2014 was used as the indicator virus in the Var IFA assay. A positive signal at a serum dilution of 1:40 or higher was considered to be IFA antibody positive.

### PEDV ELISAs for antibody detection

The U.S. PEDV prototype strain whole virus-based indirect ELISA (ProWV ELISA) was developed and validated at the ISU VDL for detection of PEDV-specific IgG antibody [15, 16]. All serum samples were tested by this ProWV ELISA following the procedures that had been previously described in detail [20]. The sample-to-positive (S/P) ratio of >0.8 was considered antibody positive, an S/P ratio between 0.6 and 0.8 was considered suspect, and an S/P ratio <0.6 was considered negative.

A previously published U.S. PEDV prototype strain S1-based indirect ELISA (ProS1 ELISA) was used to test all the serum samples in this study for the IgG antibody following the previously described procedures [14]. The S/P ratio of >0.2 was considered antibody positive, 0.14–0.2 was considered suspect, and an S/P ratio <0.14 was considered negative.

A U.S. PEDV S-INDEL-variant strain S1-based indirect ELISA (VarS1 ELISA) was developed in this study to detect the IgG antibody. The region encoding the S1 portion (aa 1–735) of the U.S. PEDV S-INDEL-variant strain was codon optimized and synthesized with the addition of a 5′ Kozac sequence, a 5′ eukaryotic signal sequence, and a 3′ 6 × -His tag by GeneArt^®^ Gene Synthesis (Thermo Fisher Scientific, Waltham, MA, USA). The resultant 2,358 base pair DNA fragment was cloned into a Zoetis proprietary eukaryotic expression vector (pZOE15). The authenticity and orientation of the insert in the recombinant plasmid was confirmed by sequencing. The recombinant plasmid was transiently transfected into human embryonic kidney (HEK) 293 cells using a Zoetis proprietary PEI transfection method. At 7 days post-transfection, culture supernatants were harvested and filter sterilized. The recombinant protein was purified via Ni-NTA Purification System (Thermo Fisher Scientific). The optimum antigen concentration and the optimum serum dilutions for the VarS1 ELISA were determined using a checkerboard titration. Polystyrene 96-well microtitration plates (Nunc^®^, Thermo Fisher Scientific) were coated (100 μl per well) with PEDV variant S1 protein and incubated overnight at 4 °C. After 5 washes with phosphate buffered saline (PBS), the plates were blocked (300 μl/well) with PBS containing 1 % bovine serum albumin (Jackson ImmunoResearch Inc., West Grove, PA, USA) for 2 h at 25 °C. Plates were dried at 37 °C for 4 h and stored at 4 °C in a sealed bag with desiccant packs until use. Serum samples were diluted 1:50 and added to the coated plates (100 μl/well). Plates were incubated at 25 °C for 1 h and then washed 5 times with PBS. Subsequently 100 μl of peroxidase-conjugated goat anti-porcine IgG (H + L) (Jackson ImmunoResearch Inc., West Grove, PA, USA) at 1:25,000 dilution was added and plates were incubated at 25 °C for 1 h. After a washing step, 100 μl tetramethylbenzidine-hydrogen peroxide substrate (TMB, Dako North America Inc., Carpinteria, CA, USA) was added. Plates were incubated at room temperature for 5 min and the reaction was stopped by adding 50 μl stop solution (1 M sulfuric acid). Reactions were measured as optical density (OD) at 450 nm using an ELISA plate reader operated with commercial software (Biotek^®^ Instruments Inc., Winooski, VT, USA). The serum antibody response was presented as sample-to-positive (S/P) ratios calculated as: S/P ratio = (sample OD – negative control mean OD)/(positive control mean OD – negative control mean OD). The PEDV VarS1 ELISA was validated using 29 field serum samples collected from a farm with documented exposure to the U.S. PEDV S-INDEL-variant strain (serum samples were collected from 29 weaned pigs one month after they were found positive for S-INDEL-variant strain by PCR) and 20 PEDV-negative field serum samples. The S/P ratio of >0.3 was considered antibody positive, 0.2–0.3 was suspect, and <0.2 was negative.

### Virus neutralization (VN) test

Serum samples were tested by a U.S. PEDV prototype strain-based VN (Pro VN) and a U.S. PEDV S-INDEL-variant strain-based VN (Var VN) following the previously described procedures [20]. The PEDV prototype isolate USA/IN19338/2013 was used as the indicator virus in the Pro VN assay and the S-INDEL-variant isolate USA/IL20697/2014 was used as the indicator virus in the Var VN assay. The reciprocal of the highest serum dilution resulting in >90 % reduction of staining as compared to the negative serum control was defined as the VN titer of the serum sample. A VN titer of ≥8 was considered positive.

### Statistical analysis

The Log_2_ (IFA titer/10) of the Pro antisera and the Var antisera tested by Pro IFA and Var IFA were analyzed in a generalized linear mixed model (GLIMMIX). Days post inoculation and antigen were used as independent variables, and pig ID and the interaction of pig ID and antigen were set as random effects. The Log_2_ (VN titer) of the Pro antisera and the Var antisera tested by Pro VN and Var VN were analyzed in a similar way. For ELISA analysis, ELISA antigen, pig ID and DPI were used as independent variables. All statistical analyses were performed with Statistical Analysis System (SAS) version 9.3 (SAS institute, Cary, NC, USA), with *p* value <0.05 considered significantly different.

## Results

### Isolation of the U.S. PEDV S-INDEL-variant strain in cell culture

Virus isolation was first attempted on 68 clinical samples received at the ISU VDL that tested positive for the U.S. PEDV S-INDEL-variant strain but virus isolation attempts in cell culture were unsuccessful. Subsequently a PEDV S-INDEL-variant strain-positive intestine homogenate was used to inoculate three 3-week-old pigs. The rectal swab of one pig had a PEDV RT-PCR Ct < 15 at 2 DPI and the pig was euthanized and necropsied at 3 DPI. The rectal swabs of the other two pigs had PEDV RT-PCR Ct < 15 at 3 DPI and both pigs were euthanized and necropsied at 4 DPI. The small intestine tissues and cecum contents collected at necropsy were used to attempt virus isolation in Vero cells. The U.S. PEDV S-INDEL-variant strain was successfully isolated from small intestine homogenates and cecum contents collected from all 3 pigs. Typical PEDV cytopathic effects including syncytial body formation and cell detachment were observed and the virus growth was confirmed by immunofluorescence staining using PEDV-specific monoclonal antibody.

One U.S. PEDV S-INDEL-variant isolate designated as USA/IL20697/2014 was selected for further propagation and characterization. This isolate was serially passed in Vero cells and the infectious titers ranged from 10^3^–10^5^ TCID_50_/ml for the first ten passages. The whole genome sequences of the isolate USA/IL20697/2014 at passage 5 (P5) had 99.3–99.9 % nucleotide identity to other U.S. PEDV S-INDEL-variant sequences available in GenBank. The S1 sequences of the USA/IL20697/2014 cell culture isolate P5 had 99.8 % nucleotide identity (only 4 nucleotide differences) to the original intestine homogenate from which the virus isolate was derived. The USA/IL20697/2014 isolate was tested at the ISU VDL and confirmed negative for TGEV, PRCV, PDCoV, porcine rotavirus A, B, C, influenza A virus, porcine reproductive and respiratory syndrome virus, and porcine circovirus 2 by virus-specific PCRs.

### Generation of antisera against the U.S. prototype and S-INDEL-variant PEDVs

The U.S. PEDV prototype isolate USA/IN19338/2013 and S-INDEL-variant isolate USA/IL20697/2014 successfully established infections in all inoculated pigs as evidenced by PCR testing of the rectal swabs. In prototype group, 4/5, 5/5, 5/5, 5/5, 5/5, 5/5, and 3/5 pigs shed the virus in rectal swabs at 2, 4, 7, 10, 14, 21, and 28 DPI, respectively, as tested by PEDV real-time RT-PCR. In S-INDEL-variant group, 3/5, 5/5, 5/5, 5/5, 4/5, 3/5 and 1/5 pigs shed the virus in rectal swabs at 2, 4, 7, 10, 14, 21 and 28 DPI, respectively. The rectal swabs of the negative control pigs remained PEDV PCR negative throughout the study period. In total, 25 antisera were collected from the prototype strain-inoculated pigs (Pro antisera), 25 antisera collected from the variant strain-inoculated pigs (Var antisera), and 25 antisera collected from negative control group (Neg antisera), at 0, 7, 14, 21, and 28 DPI.

### Evaluation of cross-reactivity of antibodies against the U.S. PEDV prototype strain and S-INDEL-variant strain by PEDV IFA antibody assays

**Fig. 1:**
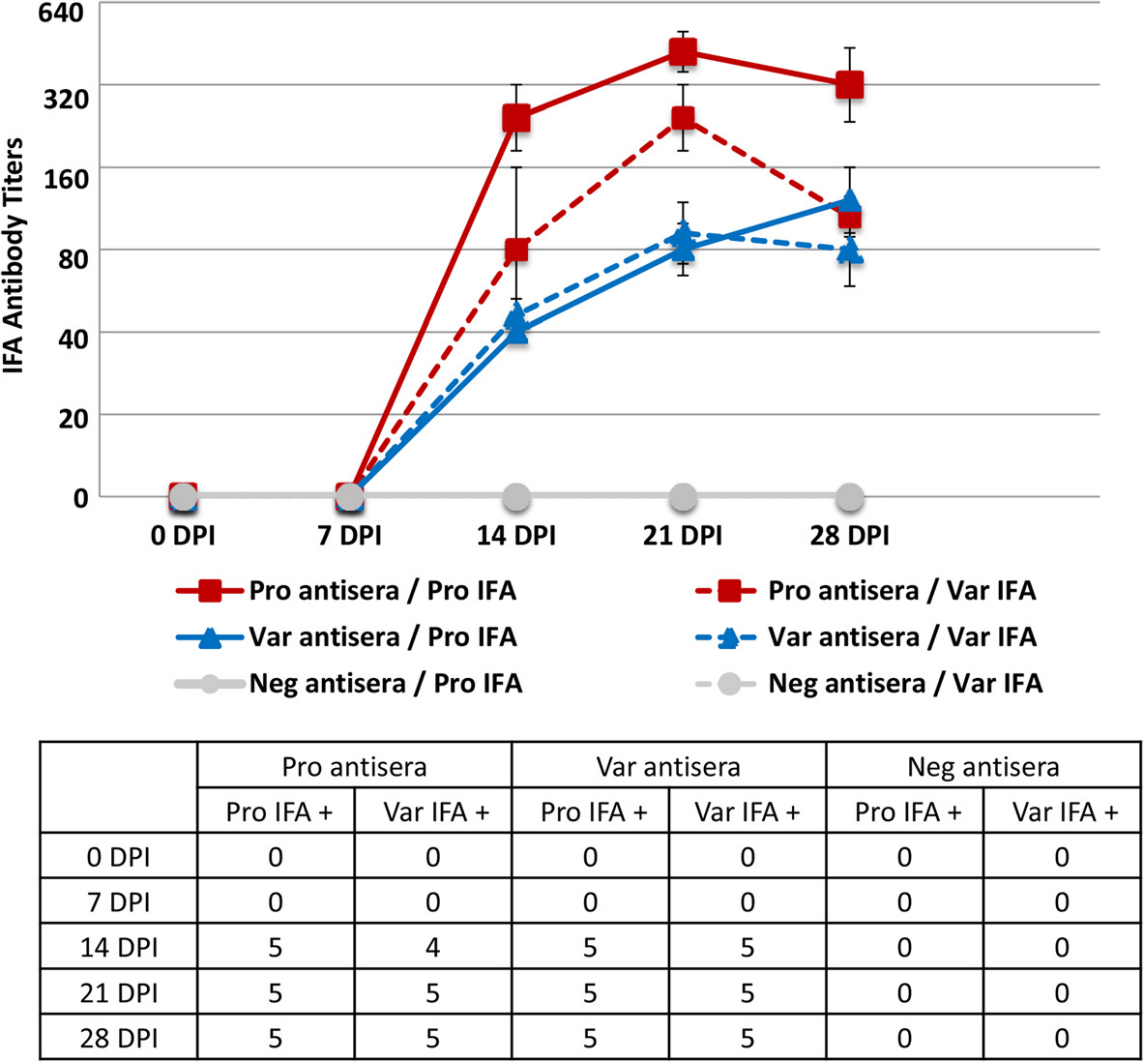
IFA antibody testing of antisera against the U.S. PEDV prototype and S-INDEL-variant strains. The average IFA antibody titers are shown at the top and the number of IFA antibody positive samples is shown at the bottom. Pro antisera: antisera collected from the U.S. PEDV prototype strain-inoculated pigs; Var antisera: antisera collected from the U.S. PEDV S-INDEL-variant strain-inoculated pigs; Neg antisera: antisera collected from negative control pigs; Pro IFA: the U.S. PEDV prototype strain-based IFA; Var IFA: the U.S. PEDV S-INDEL-variant strain-based IFA

As shown in Fig. 1, the Pro antisera tested antibody negative (0/5) at 0 and 7 DPI and 100 % positive (5/5) at 14, 21, and 28 DPI by the prototype strain-based IFA antibody assay (Pro IFA). The variant strain-based IFA (Var IFA) gave similar results on the Pro antisera except that one serum collected at 14 DPI was negative by the Var IFA assay. When the antibody titers were compared, the positive Pro antisera overall reacted better to the Pro IFA assay than to the Var IFA assay, with 1.4 log2 higher titer on average (Fig. 1).

The Var antisera tested negative (0/5) at 0 and 7 DPI and 100 % positive (5/5) at 14, 21, and 28 DPI by both the Pro IFA and Var IFA antibody assays. When the antibody titers were compared, the positive Var antisera reacted similarly to both Pro IFA and Var IFA assays, with less than 0.1 log2 titer differences on average (Fig. 1).

The antisera collected from the negative control group (Neg antisera) were antibody negative by both PEDV Pro IFA and Var IFA assays throughout the study. The pig antiserum against the European PEDV CV777 strain had similar antibody titers by the Pro IFA assay (titer 320) and by the Var IFA assay (titer 160). The antisera against TGEV Purdue, PHEV, PDCoV, and PRCV viruses were all negative by both PEDV Pro IFA and Var IFA assays.

### Evaluation of cross-reactivity of antibodies against the U.S. PEDV prototype strain and S-INDEL-variant strain by various PEDV ELISAs

**Fig. 2:**
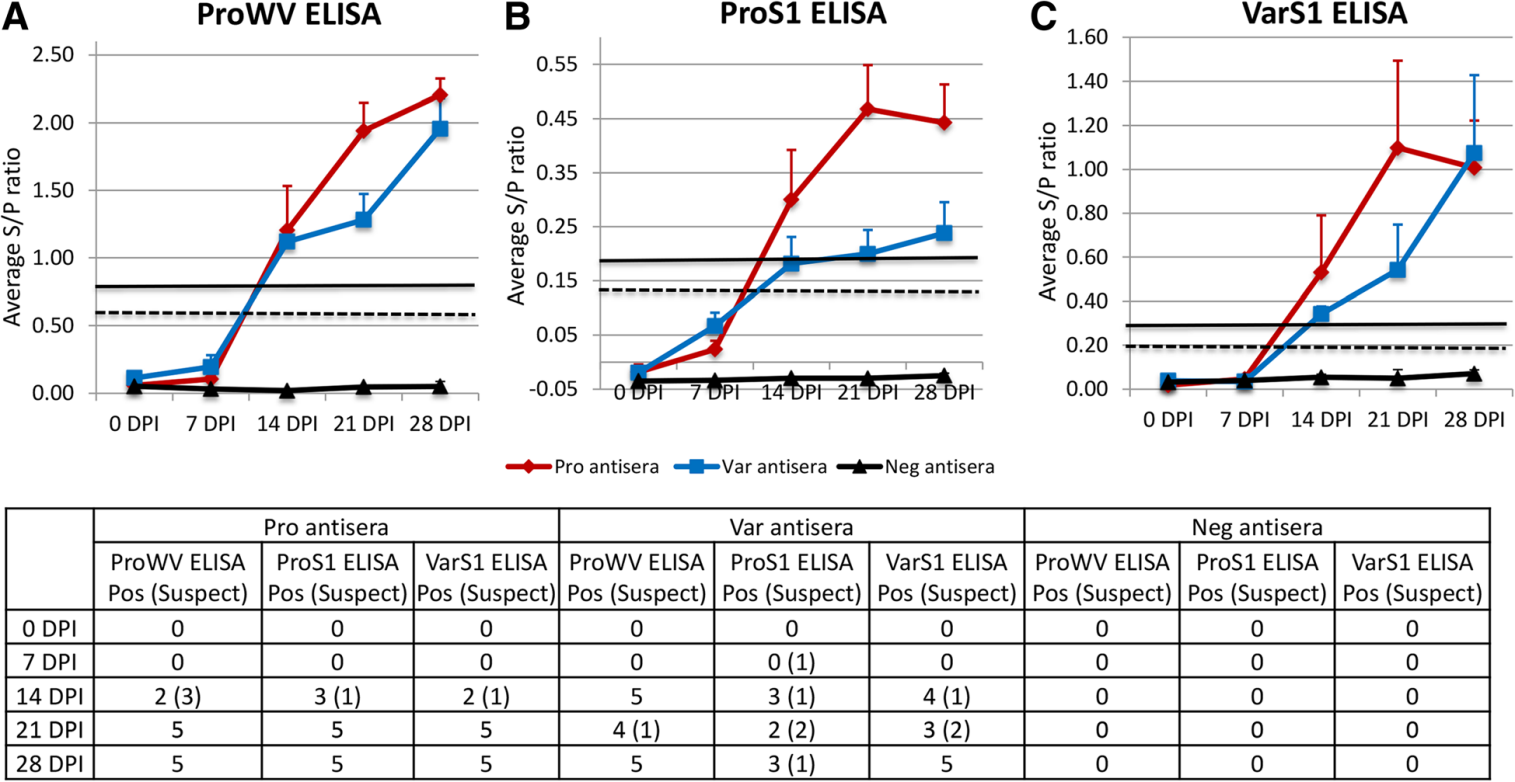
Testing of antisera against the U.S. PEDV prototype and S-INDEL-variant strains by ProWV ELISA (**a**), ProS1 ELISA (**b**) and VarS1 ELISA (**c**). For each assay, the solid black line indicates the S/P ratio above which the sample was positive; the dot black line indicates the S/P ratio below which the sample was negative; samples with S/P ratios between the solid and dot black line were suspect. ProWV ELISA: the U.S. PEDV prototype strain whole virus-based ELISA; ProS1 ELISA: the U.S. PEDV prototype strain S1-based ELISA; VarS1 ELISA: the U.S. PEDV S-INDEL-variant strain S1-based ELISA

As shown in Fig. 2, the Pro antisera collected at 0 and 7 DPI were all antibody negative by ProWV ELISA, ProS1 ELISA, and VarS1 ELISA. For the Pro antisera collected at 14 DPI, 2 sera were positive and 3 were in the suspect range by the ProWV ELISA; 3 positives and 1 suspect by the ProS1 ELISA; 2 positives and 1 suspect by the VarS1 ELISA. The Pro antisera collected at 21 and 28 DPI were all positive by three ELISAs. When comparing the total number of positive Pro antisera at 14, 21 and 28 DPI by each ELISA, there were no significant differences among three ELISAs to detect antibody against the U.S. PEDV prototype strain.

The Var antisera collected at 0 and 7 DPI were antibody negative by all three ELISAs, with the exception of one serum at 7 DPI that was in the suspect range by the ProS1 ELISA (Fig. 2). The Var antisera collected at 14, 21 and 28 DPI had variable numbers of positive, suspect and negative results by three ELISAs (Fig. 2). Overall for the Var antisera, the ProWV ELISA detected 14 sera as antibody positive, 1 as suspect, and 10 as negative; the ProS1 ELISA detected 8 sera as positive, 5 as suspect, and 12 as negative; the VarS1 ELISA detected 12 sera as positive, 3 as suspect, and 10 as negative. When comparing the total number of positive Var antisera at 14, 21 and 28 DPI by each ELISA, the ProWV ELISA was significantly better than the ProS1 ELISA to detect antibody against the U.S. PEDV S-INDEL-variant strain (*p* = 0.0079). However, there were no significant differences between the ProWV ELISA and VarS1 ELISA (*p* = 0.3643), or between the ProS1 ELISA and VarS1 ELISA (*p* = 0.0723), to detect antibody against the U.S. PEDV S-INDEL-variant strain.

The antisera collected from the negative control group (Neg antisera) were antibody negative by all three PEDV ELISAs throughout the study period 0–28 DPI. The pig antiserum against the European PEDV CV777 strain was antibody positive by all three PEDV ELISAs. The antisera against TGEV Purdue, PHEV, PDCoV, and PRCV viruses were all negative by three PEDV ELISAs.

### Evaluation of cross-neutralization of antibodies against the U.S. PEDV prototype strain and S-INDEL-variant strain by virus neutralization tests

**Fig. 3:**
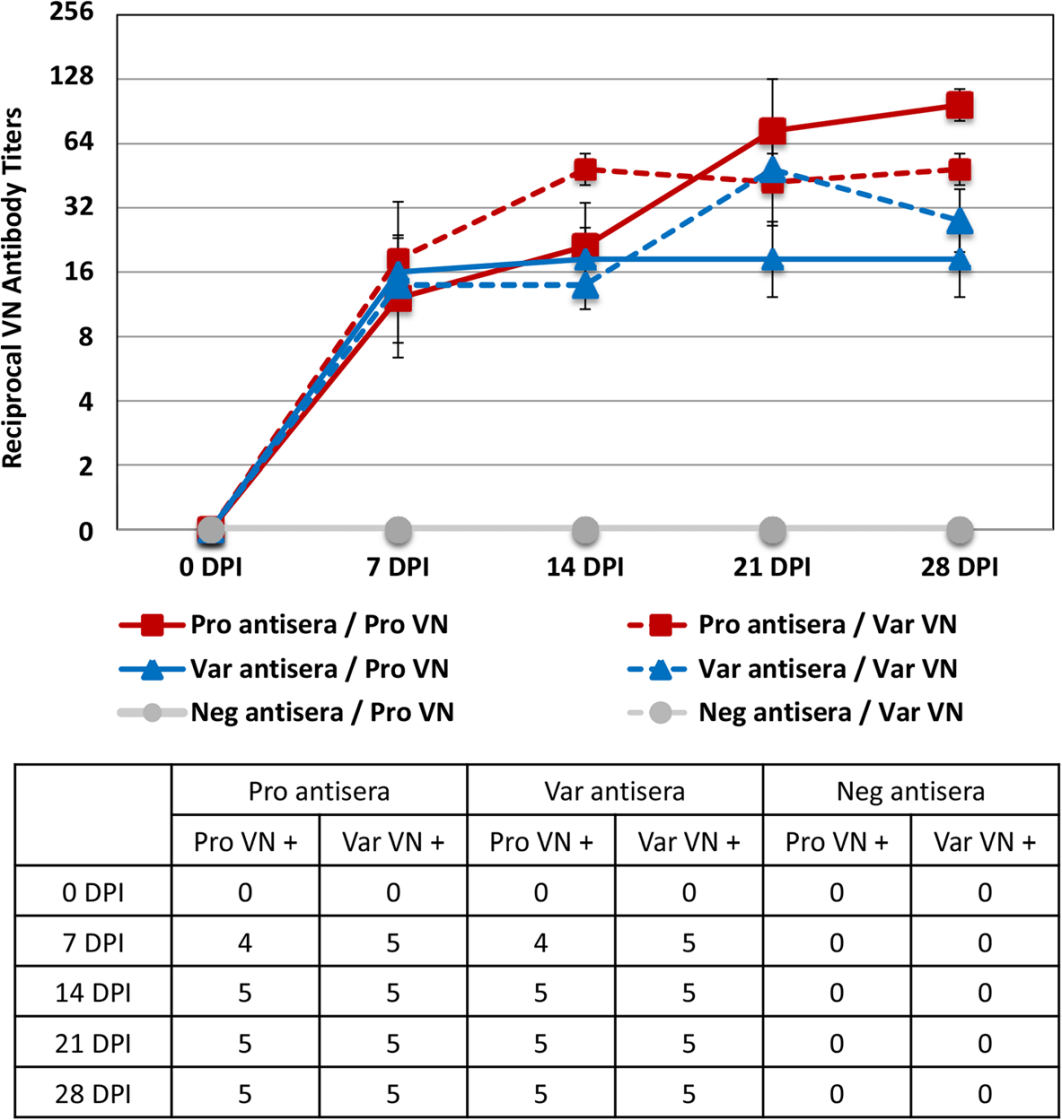
Virus neutralization antibody testing of antisera against the U.S. PEDV prototype and S-INDEL-variant strains. The average VN antibody titers are shown at the top and the number of VN antibody positive samples is shown at the bottom. Pro VN: the U.S. PEDV prototype strain-based VN; Var VN: the U.S. PEDV S-INDEL-variant strain-based VN

As shown in Fig. 3, VN antibodies were detected as early as 7 DPI in sera of most of the pigs inoculated with either a prototype strain or an S-INDEL-variant strain, regardless of testing by Pro VN or Var VN assays. Serum samples collected at 14, 21 and 28 DPI from all pigs inoculated with PEDV prototype strain or S-INDEL-variant strain were VN antibody positive by both Pro VN and Var VN assays.

The positive Pro antisera had similar VN antibody titers by the Pro VN and Var VN assays and there was no significant difference between the two assays. The positive Var antisera had similar VN antibody titers by the Pro VN and Var VN assays and overall there was no significant difference between the two assays (*p* = 0.42) although the average VN antibody titers of Var antisera at 21 and 28 DPI were slightly higher by the Var VN assay than by the Pro VN assay (Fig. 3).

The VN antibody titers of the positive Pro antisera tested by the homologous Pro VN assay were, on average, 0.8 log2 higher than the VN antibody titers of the positive Var antisera tested by the homologous Var VN assay (Fig. 3).

The antisera collected from the negative control group (Neg antisera) were antibody negative by both Pro VN and Var VN assays throughout the study period 0–28 DPI. The pig antiserum against the European PEDV CV777 strain was antibody positive by the Pro VN assay (titer 64) and by the Var VN assay (titer 16). The antisera against TGEV Purdue, PHEV, PDCoV, and PRCV viruses were all negative by both PEDV Pro VN and Var VN assays.

## Discussion

Our lab has previously isolated the U.S. PEDV prototype strains in Vero cells [7]. In order to obtain a cell culture isolate of U.S. PEDV S-INDEL-variant strains to generate strain-specific antisera for evaluation, virus isolation was first attempted in Vero cells using 68 PEDV S-INDEL-variant strain-positive clinical samples submitted to the ISU VDL. However, attempts to isolate S-INDEL-variant virus in cell culture from these samples were unsuccessful. This could be due to multiple factors such as low concentration of virus in samples, cytotoxicity of some samples, and variable storage conditions of the clinical samples after collection. Next, among the 68 clinical samples, one intestine homogenate containing the S-INDEL-variant PEDV was inoculated into pigs to generate more fresh materials with abundant virus load for virus isolation attempts in cell culture. Using this approach, U.S. S-INDEL-variant PEDV was successfully isolated in Vero cells. It is speculated that high concentration of virus in the samples and immediate virus isolation attempts on the fresh samples are the key to success of virus isolation in cell culture. For other viruses under occasions that there is difficulty to isolate those viruses in cell cultures directly from the clinical samples of naturally infected animals, the approach described in this study can be considered, namely amplifying the virus in host animals to obtain fresh samples with high concentration of virus for virus isolation attempts in cell cultures.

Some field serum samples collected from swine farms were submitted to the ISU VDL for PEDV antibody detection. However, due to the lack of clear exposure history of these cases as well as the possibility of infection with multiple pathogens or with more than one PEDV strain, these field serum samples were not ideal for evaluating serological cross-reactivity of different PEDV strains. Therefore, in the present study, antisera against the U.S. PEDV prototype and S-INDEL-variant strains were generated in weaned pigs under strict experimental conditions, for evaluation of cross-reactivity by various serological assays.

The positive antisera against the prototype strain reacted with both prototype and S-INDEL-variant viruses, and the positive antisera against the S-INDEL-variant strain also reacted with both prototype and S-INDEL-variant viruses, as examined by IFA antibody assays. When taking the antibody titers into consideration, antibodies against the prototype strain reacted better to the Pro IFA assay than to the Var IFA assay whereas antibodies against the S-INDEL-variant strain reacted similarly to the Var IFA and Pro IFA assays. Thus, the current U.S. PEDV prototype strain-based IFA antibody assay offered at veterinary diagnostic laboratories can be used to detect antibodies against both U.S. PEDV strains.

The ProWV ELISA and ProS1 ELISA have been previously developed and validated to detect PEDV-specific antibodies [14–16]. A PEDV VarS1 ELISA was developed in this study. However, this VarS1 ELISA was only validated using a limited number of field antisera against the U.S. PEDV S-INDEL-variant strain before testing the experimentally generated antisera in this study. Further validation of this VarS1 ELISA using large number of serum samples would be needed to determine the performance of this assay. All three PEDV ELISAs reacted with the Pro antisera and Var antisera. The three ELISAs detected Pro antisera similarly. However, it appeared that the ProS1 ELISA used in this study was not as efficient as the ProWV ELISA and the VarS1 ELISA to detect the antibodies against the U.S. PEDV S-INDEL-variant strain under the conditions of this study.

The antibodies against the U.S. prototype strain and the antibodies against the U.S. S-INDEL-variant strain neutralized both virus strains to similar titers. The U.S. PEDV prototype strain-based VN tests currently run in the laboratories can be used to detect antibodies against both U.S. PEDV strains.

Both the prototype and S-INDEL-variant PEDV-inoculated pigs developed detectable IFA and ELISA antibodies in sera starting from 14 DPI in this study. In contrast, both groups of pigs developed low levels of serum neutralizing antibodies starting from 7 DPI. The IFA and ELISA assays in this study detected IgG antibodies; the VN tests could potentially detect any antibody isotype with neutralizing activity. It is unclear whether this contributes to the observed early detection of low-level VN antibody. In a previous study, it has also been reported that PEDV VN antibody could be detected as early as 7 DPI [20].

The distinct genetic differences between the U.S. prototype and S-INDEL-variant PEDVs are located in the S1 region (nucleotides 1–2214 corresponding to aa 1–738, according to positions in the prototype strain USA/IN19338/2013, GenBank accession number KF650371), especially the N-terminal region of the S gene (nucleotides 1–1170 corresponding to aa 1–390) whereas the remaining portions of the genomes are relatively conserved between the two U.S. strains [4, 8, 10]. The PEDV prototype strain S1 protein used for the ProS1 ELISA and the PEDV S-INDEL-variant strain S1 protein used for the VarS1 ELISA had 92 % amino acid identity. The reported PEDV neutralizing epitopes are located in the S protein amino acid residues 499–638, 744–759, 756–771, and 1368–1374 [1, 21]. The protein sequences in these locations harboring the neutralizing epitopes are conserved between the U.S. prototype and S-INDEL-variant PEDVs. This can explain why the antibodies against the two PEDV strains were able to cross-neutralize two virus strains. The ProS1 and VarS1 ELISAs were developed using the recombinant PEDV S1 proteins (aa 1–738). Although the U.S. prototype and S-INDEL-variant PEDVs have considerable differences in aa 1–390, the two strains still have some common epitopes in this region. In addition, the recombinant S1 proteins of two PEDV strains have relatively conserved sequences from aa 390–738 including the neutralizing epitopes in this region. These may be the reasons why the ProS1 and VarS1 ELISAs can detect antibodies against both U.S. PEDV strains despite of possible differences on the sensitivity between assays. The IFA antibody assay and ProWV ELISA are supposed to detect antibodies against multiple antigenic proteins of PEDV and thus they are expected to detect antibodies against both U.S. prototype and S-INDEL-variant PEDVs. Other laboratories have developed nucleocapsid protein-based ELISAs [18] that were not evaluated in this study. Considering that the nucleocapsid protein is rather conserved among PEDVs, it is expected that the nucleocapsid protein-based ELISAs should detect antibodies against both U.S. PEDV strains. We also included one pig antiserum against the classical European PEDV CV777 strain for evaluation and the PEDV CV777 antibody was detected by all serological assays evaluated in this study. However, antisera against TGEV Purdue, PHEV, PDCoV and PRCV had no cross-reactivity with PEDV serological assays evaluated in this study.

In a previous study by Lin et al [22], hyperimmune pig antisera against U.S. PEDV prototype strain, U.S. PEDV S-INDEL-variant strain, TGEV Purdue strain, and TGEV Miller strain were generated and tested by cell culture immunofluorescence (CCIF) assay (similar to our IFA antibody assay) and fluorescent focus reduction virus neutralization (FFRVN) assay (similar to our VN test). They found that antisera against the U.S. PEDV prototype strain, S-INDEL-variant strain, and European CV777 strain all had cross-reactivity by CCIF and FFRVN assays. Our findings are consistent with their results. In addition to similar serological assays used by Lin et al, we also evaluated PEDV serological reactivity via three PEDV ELISAs. Also, we tested sequential serum samples (0–28 DPI) from pigs experimentally infected with two U.S. PEDV strains, providing useful information about the kinetics of PEDV antibody production in weaned pigs. An interesting finding in the Lin et al study was that hyperimmune antisera against TGEV Miller strain rather than TGEV Purdue strain cross-reacted with all PEDV strains by CCIF assay but not by FFRVN assay. They further demonstrated that one epitope on the N-terminal region of PEDV/TGEV N protein may contribute to this cross-reactivity. We did not include an antiserum against TGEV Miller strain in our study and could not evaluate its cross-reactivity in our PEDV assays.

## Conclusions

The data in the present study indicate that the antibodies against U.S. PEDV prototype and S-INDEL-variant strains cross-reacted and cross-neutralized both strains in vitro. The current serological assays based on U.S. PEDV prototype strain can detect antibodies against both U.S. PEDV strains. However, the cross-protection efficacy of these two PEDV strains needs to be determined by in vivo pig studies. Goede et al [23] showed that sows exposed to S-INDEL-variant PEDV infection 7 months ago could provide partial protection to newborn piglets challenged with a U.S. PEDV prototype strain. But more in vivo studies in this respect are needed to reveal whether a U.S. PEDV prototype strain or the S-INDEL-variant strain or both should be used to develop a vaccine for providing protection against both PEDV strains circulating in U.S. swine.

### Availability of supporting data

The data set(s) supporting the results of this article is included within the article.

### Ethics approval

The experimental protocols for the pig studies performed in this paper were approved by the Iowa State University Institutional Animal Care and Use Committee (approval numbers 3-14-7766-S and 6-14-7809-S).

### Consent for publication

Not applicable.

## Abbreviations

ELISA: enzyme-linked immunosorbent assay
IFA: indirect fluorescent antibody
Neg antisera: antisera collected from negative control pigs
PED: porcine epidemic diarrhea
PEDV: porcine epidemic diarrhea virus
Pro antisera: antisera collected from the U.S. PEDV prototype strain-inoculated pigs
Pro IFA: the U.S. PEDV prototype strain-based IFA
Pro VN: the U.S. PEDV prototype strain-based VN
ProS1 ELISA: the U.S. PEDV prototype strain S1-based ELISA
ProWV ELISA: the U.S. PEDV prototype strain whole virus-based ELISA
S INDEL: spike gene insertions and deletions
Var antisera: antisera collected from the U.S. PEDV S-INDEL-variant strain-inoculated pigs
Var IFA: the U.S. PEDV S-INDEL-variant strain-based IFA
Var VN: the U.S. PEDV S-INDEL-variant strain-based VN.
VarS1 ELISA: the U.S. PEDV S-INDEL-variant strain S1-based ELISA
VN: virus neutralization
